# The effects of betamethasone on clinical outcome of the late preterm neonates born between 34 and 36 weeks of gestation

**DOI:** 10.1186/s12884-021-04246-x

**Published:** 2021-11-16

**Authors:** Yas Arimi, Narges Zamani, Mamak Shariat, Hossein Dalili

**Affiliations:** 1 Resident of Obstetrics and Gynecology, Maternal Fetal Neonatal Researsh Center, Tehran University of Medical Sciences, Tehran, Iran; 2 Department of Obstetrics and Gynecology, Vali-e-Asr Hospital, Tehran University of Medical Sciences, Tehran, Iran; 3 Vali-Asr Hospital, Imam Khomeini Hospital Complex (IKHC), Keshavarz Blvd, Tehran, Iran; 4 Maternal, Fetal & Neonatal Research Center-Breastfeeding Research Center, Tehran University of Medical Sciences, Tehran, Iran; 5 Breastfeeding Research Center, Vali-e-Asr Hospital, Tehran University Of Medical Sciences, Tehran, Iran

**Keywords:** Betamethasone, Late preterm, Neonatal outcome

## Abstract

**Background:**

Prenatal corticosteroid administration in preterm labor is one of the most important treatments available to improve neonatal outcomes; however, its beneficial effects on late preterm infants (after the 34th week of gestation) remained unknown. We aimed to assess the effects of betamethasone on the clinical condition of the late preterm infants born between 34 and 36 weeks of gestation.

**Methods:**

This retrospective cohort study was performed on 100 consecutive infants born between 34 and 36 weeks of gestation and received betamethasone before delivery as the cases and 100 neonates with the same delivery conditions but without receiving betamethasone. All neonates were followed up within hospitalization to assess the neonatal outcome.

**Results:**

The neonates receiving betamethasone suffered more from respiratory distress syndrome (49% versus 31%, *p* = 0.008, RR = 1.59 95% CI (1.12–2.27)) and requiring more respiratory support (71% versus 50%, *p* = 0.002, RR = 1.43 95% CI (1.13–1.80)) as compared to the control group. There was no difference between the two groups in other neonatal adverse events or death.

**Conclusion:**

the use of betamethasone in the late preterm period (after 34 weeks of gestation) has no beneficial effects on lung maturity or preventing neonatal adverse outcomes, even may lead to increase the risk for RDS and requiring respiratory support.

**Supplementary Information:**

The online version contains supplementary material available at 10.1186/s12884-021-04246-x.

## Introduction

Improvements in maternal and neonatal health have been achieved over the past 30 years, which cannot guarantee a reduction in the incidence of preterm labor, but can cause significant changes in morbidity and fetal and neonatal survival. Premature labor is a partial common phenomenon that has been identified in 7 to 10% of all pregnant women [1]. In this condition and to maximized fetal lung development, some conservative treatments are recommended in the last weeks of pregnancy. In this regard, the administration of glucocorticoids has played a pivotal role [2]. Reducing the incidence of neonatal respiratory distress syndrome (RDS) by up to 50% after glucocorticoid administration has been shown for the first time by Liggins and Hawie [3]. Taking corticosteroids between 34 to 36 weeks of gestation promotes fetal lung development, increases neonatal Apgar score, and reduces the likelihood of RDS [4, 5]. Additionally, treatment with glucocorticoids in such pregnancies can reduce neonatal mortality and morbidity due to reducing the risk for intraventricular hemorrhage, necrotizing enterocolitis, decrease the need for intensive care unit cares, and increase the risk of sepsis in the first 48 h of neonatal life [6–8]. Human studies have also shown that betamethasone used during pregnancy can potentially affect placental function, fetal growth, hypothalamic-pituitary-adrenal axis development, and endocrine stress responses during infancy [9–12]. In the fetal period, prenatal use of betamethasone in pregnant women whose fetuses are stunted has resulted in a transient improvement in blood flow to the uterus and umbilical arteries [13]. Moreover, corticosteroids in women at high risk for preterm delivery further improve the neurodevelopmental condition of those born before 34 weeks of gestation [14].

In general, prenatal glucocorticoids are widely used in pregnancies that are prone to preterm delivery. In particular, the use of these medications was common since 1994 after a conference hosted by the National Institutes of Health, because there was strong evidence that glucocorticoids prescribed before 34 weeks of gestation in women at risk for preterm birth could reduce adverse neonatal outcomes [14]. But the effects of these drugs on late preterm infants (after the 34th week of gestation) remained unknown [15]. It was believed that after about 34 to 35 weeks of pregnancy, most fetuses develop and their survival at this age differs only 1% from that of full-term infants [16]. However, it is now clear those infants born during the late preterm have more infant and childhood problems than term infants. For this reason, this question remains unanswered whether prenatal glucocorticosteroid administration is beneficial in this population. Therefore, in this study, we decided to evaluate the effects of betamethasone administration on the clinical condition of the late preterm infants born between 34 and 36 weeks of gestation.

## Materials and methods

This retrospective cohort study was performed on 100 consecutive infants born between 34 and 36 weeks of gestation and received betamethasone before delivery as the cases and 100 neonates with the same delivery conditions but without receiving betamethasone in our tertiary hospital. All neonates were followed-up within hospitalization to assess the neonatal outcome. Including criteria are all singleton pregnancy which have spontaneous preterm delivery between 34 and 36 + 6 weeks. Multiple pregnancies, major malformations, preterm delivery for fetal or maternal indications, elective caesarian section, maternal medical complication (such as GDM or hypertension ….) and pregnancies in which glucocorticoids other than betamethasone have been prescribed were excluded from the study.

The study was conducted after approval by the Vice-Chancellor for Research and also the ethical committee at Tehran University of Medical Sciences. Neonatal information in this study was taken from the statistical population of Vali-e-Asr Hospital Neonatal Research Center located in Imam Khomeini Hospital between 2017 and 2019. All neonates were followed-up within hospitalization to assess the study variables including gestational age at delivery, Apgar score of the first and fifth minute of birth, and the occurrence of neonatal complications including RDS, Infantile transient tachypnea of the newborn (TTN), apnea, intraventricular hemorrhage (IVH), necrotizing enterocolitis (NEC), neonatal sepsis, hypoglycemia, needing continuous positive airway pressure (CPAP) or respiratory support, surfactant use, length of hospital stay, and also neonatal death. The study endpoint was to assess and compare the pointed sequels in the two groups of neonates with and without receiving betamethasone.

For statistical analysis, results were presented as mean ± standard deviation (SD) for quantitative variables and were summarized by frequency (percentage) for categorical variables. Continuous variables were compared using the t-test or Mann-Whitney test whenever the data did not appear to have normal distribution or when the assumption of equal variances was violated across the study groups. Categorical variables were, on the other hand, compared using the chi-square test. For the statistical analysis, the statistical software SPSS version 23.0 for windows (IBM, Armonk, New York) was used.

## Results

Baseline characteristics in the two groups of neonates receiving and not receiving betamethasone are shown in Table 1. The two groups were matched for baseline variables including gender, average anthropometric parameters including body weight, height, head circumference, and gestational age at delivery.

**Table 1:** Baseline characteristics in the two groups of neonates receiving and not receiving betamethasone

Characteristics	With betamethasone (*n* = 100)	Without betamethasone (*n* = 100)	*P* value
Male gender, %	54 (54.0)	53 (53.0)	0.998
Mean weight, g	2460.3 ± 532.3	2653.3 ± 581.1	0.556
Mean height, cm	43.1 ± 5.5	48.1 ± 3.8	0.224
Mean head circumference, cm	32.4 ± 2.4	33.4 ± 2.3	0.789
Gestational age at delivery-no (%)			0.60
34 w 0 days to 34 w 6 days	21 (21)	25 (25)	
35 w 0 days to 35 w 6 days	33 (33)	27 (27)	
36 w 0 days to 36 w 6 days	46 (46)	48 (48)	

With regard to neonatal consequences and outcomes (Table 2), it was found no difference between the two groups of neonates in the prevalence rate of some neonatal complications including TTN, neonatal apnea, NEC, sepsis, IVH, hypoglycemia, requiring neonatal resuscitation, PPV or CPAP, tracheal intubation, needing surfactant use, asphyxia, or Apgar score. However, those subjects receiving betamethasone suffered more from RDS (49% versus 31%, *p* = 0.008, RR = 1.59 95% CI (1.12–2.27)) and requiring more respiratory support (71% versus 50%, *p* = 0.002, RR = 1.43 95% CI (1.13–1.80)) as compared to the control group. Neonatal death occurred in 3 and 5% of the cases with and without betamethasone use respectively with no significant difference (*p* = 0.470, RR =0.60 95% CI (0.14–2.44)).

**Table 2:** Neonatal outcome in the two groups of neonates receiving and not receiving betamethasone

Characteristics	With betamethasone (*n* = 100)	Without betamethasone (*n* = 100)	*P* value	RR 95% CI
TTN, %	6 (6.0)	3 (3.0)	0.299	0.96 (0.91–1.02)
Neonatal apnea, %	2 (2.0)	0 (0.0)	0.153	0.98(0.95–1.00)
NEC, %	0 (0.0)	0 (0.0)	N/A	N/A
Neonatal sepsis, %	37 (3.0)	43 (43.0)	0.418	0.86 (0.61–1.22)
IVH, %	20 (20.0)	14 (14.0)	0.245	1.42 (0.76–2.66)
Hypoglycemia, %	10 (10.0)	10 (10.0)	1.000	1.16(0.91–1.09)
Neonatal resuscitation, %	62 (62.0)	53 (53.0)	0.198	1.16(0.92–1.48)
Respiratory support, %	71 (71.0)	50 (50.0)	0.002	1.43 (1.13–1.80)
Needing PPV, %	31 (31.0)	31 (31.0)	1.000	1.01 (0.66–1.52)
Needing CPAP, %	37 (37.0)	31 (31.0)	0.343	1.20(0.81–1.77)
Tracheal intubation, %	6 (6.0)	9 (9.0)	0.432	0.66(0.24–1.80)
RDS, %	49 (49.0)	31 (31.0)	0.008	1.59(1.12–2.27)
Needing surfactant use, %	27 (27.0)	19 (19.0)	0.166	1.43(0.85–2.40)
Asphyxia, %	1 (1.0)	0 (0.0)	0.314	0.99(0.97–1.00)
Length of hospital stay, day	9.5 ± 1.2	9.8 ± 1.3	0.114	N/A
First minute Apgar	7.0 ± 2.1	7.2 ± 1.9	0.092	N/A
Fifth minute Apgar	8.7 ± 1.1	8.7 ± 1.2	0.644	N/A
Neonatal death, %	3 (3.0)	5 (5.0)	0.470	0.60(0.14–2.44)

*TTN* transient tachypnea of the newborn, *NEC* necrotizing enterocolitis, *IVH* intraventricular hemorrhage, *PPV* positive pressure ventilation, *CPAP* continuous positive airway pressure, *RDS* respiratory distress syndrome

## Discussion

Prenatal corticosteroid administration in preterm labor is one of the most important treatments available to improve neonatal outcomes. For women who are at risk for preterm birth between 24 and 34 weeks, prenatal corticosteroid treatment is recommended. Today, the recommendation extends from 34 to 36 weeks and 6 days at risk of preterm delivery, however, its beneficial effects after 34 weeks of gestation remains uncertain. As shown in the present survey, administration of corticosteroids after 34 weeks of gestation not only is not beneficial but also may lead to even poorer neonatal outcomes by increasing the likelihood of RDS and needing respiratory supports. However, it should be noted that the effect of corticosteroid therapy may vary depending on the cause of preterm delivery, the mood of delivery, maternal factors, and different protocols of corticosteroids used. In this regard, some animal and human-based studies could confirm the beneficial effects of corticosteroids, but some others did not recommend such regimens due to their related adverse consequences. Some studies showed that the use of such regimens reduced birth weight as well as increase the risk for hypoglycemia in preterm infants. Another major concern with cases that have been exposed to corticosteroids during pregnancy was the likelihood of developing cardiovascular complications and metabolic effects, especially at older ages. In a study by Gyamfi-Bannerman et al. [17] on premature singleton pregnancies after 34 weeks, infants whose mothers received corticosteroids had a higher need for respiratory support and resuscitation in the delivery room, as well as their neonates experienced more rates of RDS and TTN as similarly revealed in the present study. In another clinical trial by Gyamfi-Bannerman et al. [18] that examined the effect of corticosteroids on late preterm deliveries, secondary outcomes such as respiratory problems and the need for surfactant were significantly lower in the betamethasone group but the incidence of hypoglycemia was higher in the betamethasone group and there was no significant difference in the need for CPAP and NICU hospitalization between the two groups. In another study by Ontela et al. [19], the effect of prenatal dexamethasone administration on respiratory problems in infants born in late preterm deliveries was assessed by conducting a randomized clinical trial and concluded that prenatal dexamethasone injection did not reduce the rate of respiratory problems in infants with delayed preterm birth. In their study, the incidence of respiratory problems was even higher in the exposure group, but this difference was not significant. In a prospective study of respiratory problems in delayed preterm labor by Shaikh et al. [20], the rate of RDS or other respiratory problems did not significant across the two groups receiving or not receiving prophylactic corticosteroid. A review study by Kamath-Rayne et al. [14] examined the effects of prenatal corticosteroids after 34 weeks and concluded that although this treatment is effective in preventing respiratory problems in preterm infants, it has the greatest effect on the incidence of transient neonatal tachypnea, as a self-limiting disease. Thus this treatment, apart from respiratory problems, did not eliminate other problems of premature infants. Another review by Groom et al. [21] found that there was ample evidence of beneficial effects of prenatal corticosteroid use in pregnancies after 34 weeks or later in women with diabetes and women scheduling cesarean section that ultimately reduce respiratory problems, but the lack of information about the possible harms of this treatment such as controlling blood sugar need to more investigated in further in clinical trials. Because of the above, many studies have been performed on the administration of prenatal corticosteroids in preterm labor after 34 weeks, some of which were concluded similar to our study that the incidence of RDS and the need for respiratory support in a group who received corticosteroids before birth was higher than the control group, some did not find significant differences in any of the variables between the two groups and some others have shown that the rate of respiratory problems in the group receiving prenatal corticosteroids is significantly lower than in the control group and that corticosteroids cause lung maturation even during late preterm. Therefore, according to our study, prenatal betamethasone is not recommended in late preterm deliveries after 34 weeks of gestation.

This study has an innate limitation of being conducted as a retrospective study. We also emphasis that perspective study on more number of patients maybe in a multicenter study is needed.

## Conclusion

It can be finally concluded that the administration of betamethasone in the late preterm period (after 34 weeks of gestation) has no beneficial effects on lung maturity or preventing neonatal adverse outcomes, even may lead to increase the risk for RDS and requiring respiratory support.

## Supplementary Information

**Additional file 1.**

## Data Availability

All data generated or analysed during this study are included in this published article [and its supplementary information files].

## Abbreviations

RDS: Respiratory distress syndrome
TTN: Transient tachypnea of the newborn
IVH: Intraventricular hemorrhage
NEC: Necrotizing enterocolitis
CPAP: Continuous positive airway pressure
SD: Standard deviation
PPV: Positive pressure ventilation
NICU: Neonatal Intensive Care Unit
